# Supplementary material for: Exceptional long-term sperm storage by a female vertebrate
Source: PLoS One. 2021 Jun 4;16(6):e0252049. doi: 10.1371/journal.pone.0252049 (PMC8177532; doi:10.1371/journal.pone.0252049)
Supplement: Attachment — Submitted filename: Response to reviewers.docx [file pone.0252049.s001.docx]

**Response to Reviewer comments:**

**We thank the reviewers for the constructive feedback and have taken these into account in our revision of the manuscript. These revisions, albeit minor, enhance the quality of the manuscript.**

Reviewer #1: The authors report on a wild-collected female Crotalus atrox, maintained in isolation from 1999, that produced two healthy litters approximately one and six years post capture. Genetic analysis of the second litter identified paternal contribution in all offspring, thus rejecting facultative parthenogenesis. They suggest the long-term sperm storage to last approximately 71 months, making it the longest period over which a female vertebrate has been shown to store sperm that resulted in the production of healthy offspring from LTSS.

This is an observational study reporting an interesting finding with implications for sperm competition, mating and reproductive strategies in a snake. The methods applied and the data obtained appear valid. I have only a very few minor points that have to be clarified during a revision of the paper.

Material and methods:
I am missing a note on the approval for the collection of the animal from the wild as well as a licence for keeping it in captivity.

**Response – Information regarding the collection permits and IACUC approval are already provided in the Ethics Statement section of the manuscript.**

Whole genomic DNA was obtained from offspring: Please explain how this was done. From which tissues? Were the animals killed? By which means? And, again, under which license?

**Response – Relevant information has been included in the methods section and the lACUC protocol number can be found in the Ethics Statement.**

Discussion:
Appears rather long. Please shorten if possible.

**Response – While reviewer 1 suggests shortening the discussion, reviewer 2 does not, and but instead suggests expansion of the section discussing the anatomical mechanisms of sperm storage in snakes. In light of recent studies which report uterine muscular twisting in gravid females (a finding which contradicts the function of this anatomical process in LTSS), our report of sequential LTSS litters despite isolation from males suggest that this mechanism is indeed important in LTSS, and may actually permit the storage of sperm over multiple reproductive events. As such, we feel that this, in concert with the significance of our findings to sexual selection, justifies following the recommendation of reviewer 2.**

Reviewer #2: This is manuscript is well written and provides unprecedented data on exceptional long-term sperm storage for 71 months in a female Western Diamond-backed Rattlesnake (Crotalus atrox). The methods are soundly carried out. Descriptions are detailed and well documented. The combination of data obtained in captivity, genetic screening and the observation of paternal alleles in all offspring showed a very accurate result, confirming the differentiation between long-term sperm storage (LTSS) and facultative parthenogenesis (FP). Both males and females were born, all in good condition and no anomalies present, reinforcing the results presented in the paper. Some intriguing questions about long-term sperm storage remain uncertain: we still need to understand how spermatozoa are able to survive during this period and how females may produce multiple clutches using these stored sperm. The authors of this study are experts on this subject and this manuscript is a huge contribution to the knowledge on the reproductive biology of vertebrates. This is a good paper and I look forward to see this paper published. Minor suggestions are included below:

INTRODUCTION

Line 27-29: The authors mention in these lines that ectothermic vertebrates (non-avian reptiles) may show sperm storage for several years, however they do not mention where sperm is stored. A brief anatomical description of sperm storage sites in the oviducts of female reptiles, mainly for snakes is important. Later, the authors mention in the discussion section, sperm storage in UMT (uterine muscular twisting) in the útero-vaginal junction and in the infundibular portion. The inclusion of this information in the introduction will contribute to a general view on sperm storage in the oviduct, contributing to a better understanding of this reproductive tactic by the readers.

**Response – a brief statement has been added, which is expanded in the discussion.**

MATERIAL AND METHODS
The dataset and analysis presented by the authors strongly support the goals of the study, reflecting huge experience of the authors within these techniques. The methodology is well detailed and the replication of this study is possible. On line supporting information contains the protocols and algorithms used in this study, allowing their application in future research.

Line 121: Please, include the meaning of SNPs in the first time that this abbreviation appears in the text.

**Response - done**


DISCUSSION

This section is short, however interesting and well written. However, I suggest the inclusion of some recent publications on uterine muscular twisting (UMT) and infundibular sperm storage in Viperidae snakes to improve this section.

Line 217-219: This paragraph may be improved with the inclusion of these recent publications (Silva et al 2019; Silva et al., 2020, Barros et al., 2020; Souza and Almeida-Santos, 2020) on sperm storage in Bothrops and Lachesis (closely related taxa). Recent data show that the UMT does not prevent the passage of spermatozoa to the infundibulum after mating as it previously hypothesized for some Viperidae snakes. The UMT has been described in vitellogenic, non-vitellogenic and pregnant females, with and without the presence of sperm, raising many doubts about the function of this structure. Recent findings showed that sperm storage in Bothrops and Lachesis occur in the furrows of the útero-vaginal junction or in the UMT, and in infundibular sperm receptacles. It suggests that Crotalus may also have infundibular sperm receptacles.

**Response – The section has been expanded and additional citations included.**

REFERENCES
This reference was not cited in the manuscript
52 -Purcell S, Neale B, Todd-Brown K, Thomas L, Ferreira MAR, Bender D, Maller J, Sklar P, de Bakker PIW, Daly MJ, Sham PC. 2007 PLINK: a toolset for whole-genome association and population-based linkage analysis. A. J. Hum. Genet. 81, 559–575.

**Response – Incorrectly numbers as 47, but now corrected.**
